# Larger pollen loads increase risk of heat stress in foraging bumblebees

**DOI:** 10.1098/rsbl.2022.0581

**Published:** 2023-05-17

**Authors:** Malia Naumchik, Elsa Youngsteadt

**Affiliations:** Department of Applied Ecology, North Carolina State University, Raleigh, NC 27695, USA

**Keywords:** *Bombus*, critical thermal maximum, heat stress, foraging behaviour, thoracic temperature, climate change

## Abstract

Global declines in bumblebee populations are linked to climate change, but specific mechanisms imposing thermal stress on these species are poorly known. Here we examine the potential for heat stress in workers foraging for pollen, an essential resource for colony development. Laboratory studies have shown that pollen foraging causes increased thoracic temperatures (*T*_th_) in bees, but this effect has not been examined in bumblebees nor in real-world foraging situations. We examine the effects of increasing pollen load size on *T*_th_ of *Bombus impatiens* workers in the field while accounting for body size and microclimate. We found that *T*_th_ increased by 0.07°C for every milligram of pollen carried (*p* = 0.007), resulting in a 2°C increase across the observed range of pollen load sizes. Bees carrying pollen were predicted to have a *T*_th_ 1.7–2.2°C hotter than those without pollen, suggesting that under certain conditions, pollen loads could cause *B. impatiens* workers to heat from a safe *T*_th_ to one within the range of their critical thermal limits that we measured (41.3°C to 48.4°C). Bumblebees likely adopt behavioural or physiological strategies to counteract the thermal stress induced by pollen transport, and these may limit their foraging opportunities as environmental temperatures continue to increase.

## 1. Introduction

Around the world, insect populations are declining, with climate change implicated as one of the main causes [1]. Bumblebees are one of the clearest examples of climate impacts on insects, with several species experiencing severe reductions in their populations and ranges due to warming [2,3]. These losses are expected to worsen under future conditions, with the most pronounced declines predicted in agricultural areas [4]. Because bumblebees are key pollinators in natural and agricultural systems where they occur, continuing declines in this group will have extensive ecological and economic consequences [5,6].

Although bumblebees are classical models of insect thermal biology [7], specific mechanisms by which climate limits bumblebee colony development and reproduction remain unknown [8]. Successful bumblebee colony development depends on the quantity and quality of pollen [9], but the act of collecting pollen may increase bumblebees' risk of overheating. Prior laboratory studies suggest that carrying a pollen or nectar load increases metabolism in honeybees and bumblebees [7,10], probably more so for higher-quality resources [11,12]. Elevated metabolism may then increase insect thoracic temperature (*T*_th_); indeed, in queen bumblebees, heavier nectar loads correlated with hotter *T*_th_ in the laboratory [7]. Pollen transport may be even more energetically costly than nectar transport because pollen is carried with a lower centre of gravity, creates drag and does not contribute to evaporative cooling [13]. In honeybees, workers with full pollen loads had hotter *T*_th_ than those with full nectar crops [13].

Despite this evidence, it is not known whether increases in metabolism or *T*_th_ in controlled environments translate to increased risk of heat stress in the field. Moreover, effects of pollen load weight on bumblebee *T*_th_ have not been examined in any setting. We predict that bumblebee *T*_th_ and risk of heat stress increase with pollen load size. We test this prediction in the field using a common, economically important North American bumblebee.

## 2. Material and methods

### (a) Study species

We focused on worker bees of *Bombus impatiens*, the Common Eastern Bumble Bee. This is an abundant, native pollinator in the eastern United States that is also used commercially in agriculture throughout the country. Although this bee is not currently declining due to climate change [3], it is often used as a model species in bumblebee studies because its abundance and long flight season facilitate research.

### (b) Bee thoracic temperatures and sample collection

We collected samples at the JC Raulston Arboretum in Raleigh, North Carolina, USA (35.7942, −78.6981) from 12 August to 6 September 2021, between 09.00 and 14.00. We caught *B. impatiens* workers with a range of pollen load sizes. We only sampled bees that were actively foraging for pollen, to reduce interference from any added weight from nectar. Bees tend to specialize in either pollen or nectar collection on a single trip, so pollen foragers were unlikely to have full crop loads [14]. To measure each bee's internal *T*_th_, we captured it into a ‘bee squeezer’ [15] and inserted a type K thermocouple (Hyp-0, 0.2 mm diameter, attached to HH-25U reader, Omega Engineering, Norwalk, CT, USA) into the ventral thorax centred between the legs within 10 s of capture [7]. We then anaesthetized the bee with CO_2_ and removed pollen from both corbiculae using a toothpick (The Doctor's BrushPicks, Prestige Consumer Healthcare, Lynchburg, VA, USA). We then placed the bee and pollen into separate pre-dried, pre-weighed vials, stored them at −20°C, and thawed them in a desiccator prior to weighing each sample to the nearest microgram (pollen) or 0.01 mg (bees).

### (c) Operative temperatures

To isolate effects of pollen loads on bee *T*_th_, we needed to account for effects of the microclimate in which bees were foraging. Operative temperature (*T*_e_) is a species-specific metric of microclimate that represents the temperature of an organism at equilibrium with its environment. It integrates effects of air temperature, surface temperature, solar radiation, wind speed and the organism's body shape, colour and texture, in the absence of behavioural thermoregulation, metabolic heating or evaporative cooling [16]. We used a physical model to measure *T*_e_ every 2 min while we collected *T*_th_ measurements in 2021. Our operative temperature model was a dead, dried bee with a Type T thermocouple (HYP-2, with HH520 logger, Omega Engineering) inserted into its thorax. We positioned the dead bee in a life-like position on a flower near where our live bee samples were collected (electronic supplementary material, figure S1).

### (d) Critical thermal limits

The critical thermal maximum, or CT_max_, is a measure of the upper thermal limits of an organism [17]. To determine the CT_max_ of *B. impatiens*, we collected live bees foraging on flowers at 12 study sites in Raleigh and Durham, NC, USA in June to July 2022, between 08.00 and 16.00, as part of a larger project (details are provided in electronic supplementary material, text S1). Briefly, we provided bees with 1 M sucrose solution and brought them to the laboratory, where we placed them individually into 7 ml glass vials in a dry bath (IC25XT, Torrey Pines Scientific, Carlsbad, CA, USA) set at 36°C for 10 min. We then increased the temperature 1°C every 4 min, comparable to the 0.25°C min^−1^ rate used in similar studies [18–20] (electronic supplementary material, table S3). We monitored bees every 4 min until each individual reached CT_max_, which we described as the onset of spasms [17]. The temperature at which the bees reached their CT_max_ was measured with a type-K thermocouple probe (Hyp-0, 0.2 mm diameter, with HH520 logger, Omega Engineering) placed into an empty 7 ml glass vial in the dry bath. We completed CT_max_ measurements within 4 h of field collection and kept control bees in identical vials at room temperature during the assays. All bees were then dried (55°C, 48 h) and weighed to the nearest 0.01 mg.

### (e) Statistical analyses

To test for the effect of pollen weight on bees' *T*_th_, while accounting for *T*_e_ and body weight, we used multiple linear regression. We included body weight in the model because larger bees tend to be hotter [21], and *B. impatiens* workers have a large size variation. We fit the model using the ‘stats’ package in R v. 3.5.1 [22] and tested significance using a Type 3 ANOVA in the ‘car’ package [23].

To contextualize the effects of pollen load size on potential heat stress, we used our regression model to predict *T*_th_ of small, medium, and large bees, with and without pollen, relative to *B. impatiens*' CT_max_. To represent small, medium and large bees, we used the 2.5th, 50th and 97.5th percentiles of observed bee weights in the *T*_th_ dataset. Pollen loads were set to either (a) maximum observed load (32.192 mg), or (b) maximum observed per cent of bee wet weight (28.552%), which is below the maximum body weight percentage of pollen that large bees can carry [24]; we used whichever value was smaller (table 1). We used the hottest observed *T*_e_ in our dataset (55.8°C) to represent a moderately hot day in our study area; this measurement occurred when the air temperature was 30.6°C (see also electronic supplementary material, figure S4). We used quantile regression to analyse the relationship between CT_max_ and body weight and estimate the range of CT_max_ values relevant to small, medium, and large bees in the prediction scenarios (electronic supplementary material, text S1).

**Table 1.** Model inputs and predicted body temperatures of *Bombus impatiens* workers with different body sizes and pollen loads.

bee size class	bee weight (mg)	pollen weight (mg)	*T*_e_ (°C)	predicted *T*_th_ (°C)	s.e.
small	88.49	0	55.8	40.8	0.7
small	88.49	25.266	55.8	42.5	0.7
medium	168.35	0	55.8	41.9	0.5
medium	168.35	32.182	55.8	44.1	0.7
large	235.98	0	55.8	42.9	0.7
large	235.98	32.182	55.8	45.0	0.8

## 3. Results

**Figure 1.**
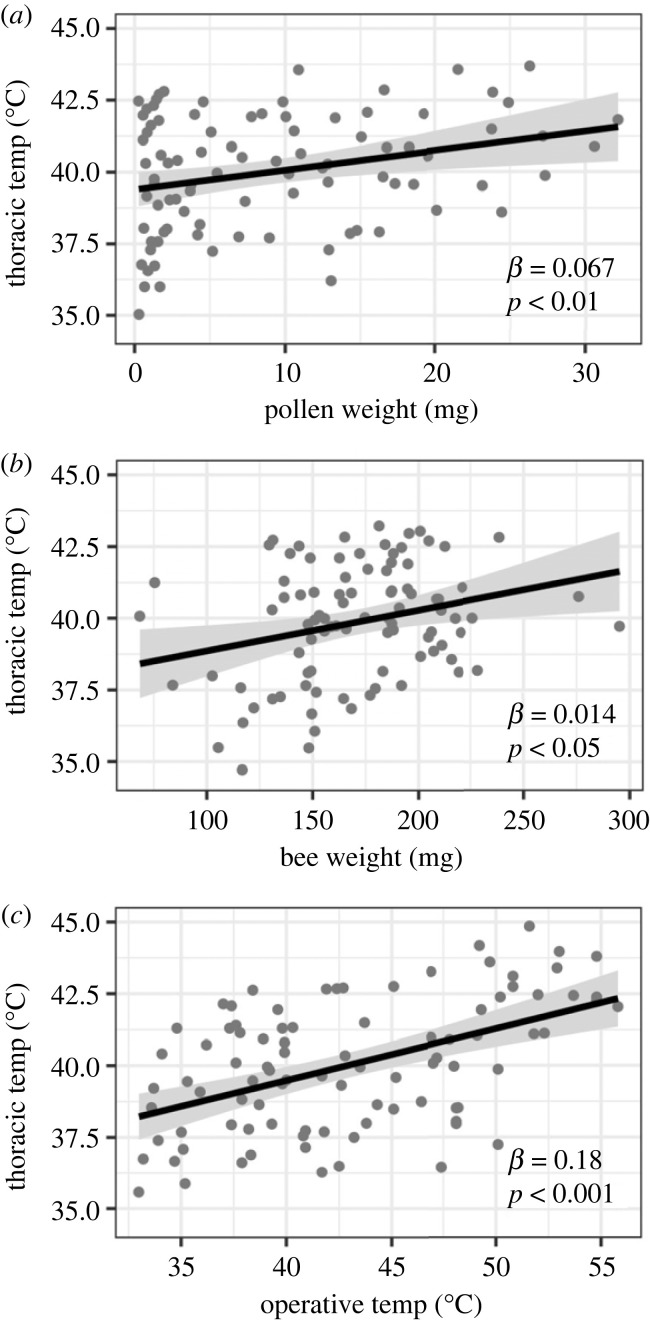
The effect of (*a*) pollen weight, (*b*) bee body weight and (*c*) operative temperature on bee thoracic temperature. Each plot illustrates the partial regression of thoracic temperature on a single predictor after accounting for the other two predictors. Each symbol represents one measurement (*n* = 91), lines are model predictions, and shaded areas are standard errors.

We collected 91 *B. impatiens* workers ranging in size from 68.51 mg to 295.17 mg, carrying pollen loads of 0.28 to 32.18 mg. Our operative temperature model recorded temperatures up to 55.8°C, while our hottest recorded bee temperature was 44.1°C. Pollen weight, bee weight and *T*_e_ were all significant predictors of bee *T*_th_ (table 2, figure 1). When controlling for bee weight and operative temperature, *T*_th_ increased 0.07°C mg^−1^ of pollen carried (figure 1*a*). Overall, the *R*^2^ of our full model was 0.299.

**Table 2.** ANOVA table and estimated coefficients ± SE for the regression model predicting *B. impatiens* thoracic temperature as a function of bee weight, pollen weight and operative temperature.

	d.f.	SS	*F*	*p*	coefficient ± s.e.
intercept	1	975.8	250.3		29.46 ± 1.86
bee weight	1	26.9	6.9	0.010	0.014 ± 0.005
pollen weight	1	29.8	7.6	0.007	0.067 ± 0.024
operative temp	1	108.5	27.8	< 0.001	0.180 ± 0.034
residuals	87	339.1			

**Figure 2.**
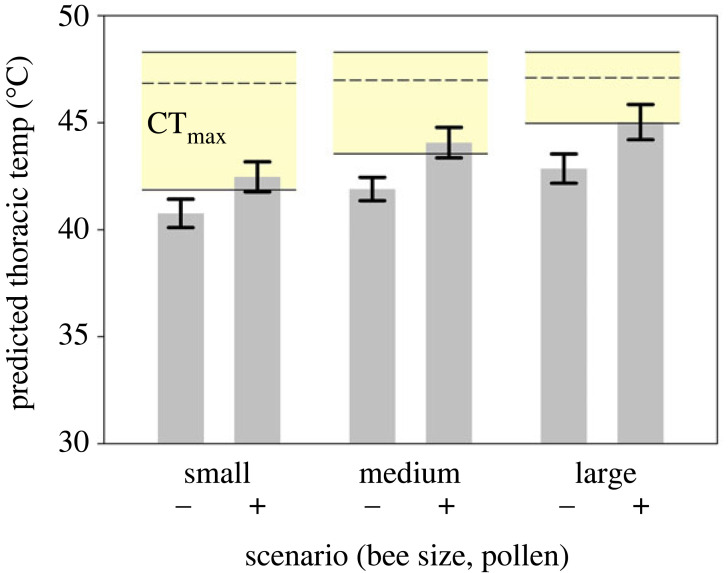
The predicted thoracic temperatures (mean ± s.e.) of small, medium and large *Bombus impatiens* workers with (+) and without (−) pollen, relative to CT_max_ values for this species (dashed line indicates the median, coloured bands indicates middle 95% of CT_max_ values for bees of the specified size).

We measured CT_max_ for 91 *B. impatiens* workers. No control bees (*n* = 20) died during CT_max_ assays. Across individuals, CT_max_ ranged from 41.3°C to 48.4°C, with a median of 47.1°C and mean of 46.6°C (s.d. = 1.5). The median CT_max_ was consistent across bees of all body weights, but low values were more common among smaller bees (electronic supplementary material, text S1, table S1, figure S2). Model predictions suggest that bees carrying pollen would all be at risk of reaching their CT_max_ under certain foraging conditions, regardless of body weight (figure 2). Bees carrying pollen were predicted to have a *T*_th_ 1.7–2.2°C hotter than those without pollen, putting their T_th_ within the range of CT_max_ values that we estimated for each size class (figure 2, table 1).

## 4. Discussion

Bumblebees are established models of thermal biology, well known for their ability to generate and conserve heat in cold conditions and dissipate it under heat stress [7,25]. However, bumblebees are declining under climate change [2,3], suggesting that their thermal toolkit cannot compensate for chronically warmer conditions. Our results highlight one set of conditions under which bee thermoregulation may be challenged in the field, with the risk of heat stress increasing with pollen load size. Specifically, *T*_th_ increased 0.07°C mg^−1^ of pollen, resulting in about a 2°C total effect on *T*_th_ across the observed range of pollen load sizes. This increase in *T*_th_ could push *B. impatiens* workers to their critical limits, as many bees were already foraging with *T*_th_ within a few degrees of their CT_max_. Although we did not examine why pollen load size affects *T*_th_, increased activity of flight muscles to generate additional lift [7,25] and increased exposure time to heat with limited resources for evaporative cooling may contribute to this effect [13].

While pollen load size clearly affected *T*_th_, several other covariates were important in our model. *T*_e_ was the strongest predictor of *T*_th_. *T*_e_ is a heat index that integrates environmental forces acting on a bee's body temperature. The realized bee body temperatures, measured as *T*_th_, then integrate the effects of *T*_e_, behaviour and metabolism. *T*_th_ increased 0.18°C per 1°C increase in *T*_e_, resulting in about a 4°C total increase of *T*_th_ over a 22.8°C range of observed *T*_e_. Despite heat generation from bees' flight muscles, 60% of the *T*_th_ measurements were cooler than the simultaneous *T*_e_ readings, likely due to the effects of evaporative and convective cooling during flight [26,27]. Bee body weight also contributed to *T*_th_, although the effect was relatively weak. There was a 0.01°C increase in *T*_th_ per milligram of body weight, with a total increase of about 2°C in *T*_th_ over the observed 227 mg range of body sizes. This finding is consistent with known effects of body size, wherein larger bees generate more heat and lose it more slowly due to lower surface area-to-volume ratios [21,24]. Although pollen load size, *T*_e_, and body weight were all strong predictors of *T*_th_, our full model had an *R*^2^ of 0.299, leaving the majority of variation in *T*_th_ unexplained. Given that we did not control for bee genetic background, age, motivation, foraging trip duration or distance or floral host, this large amount of unexplained variation is not surprising. We also found that the variability of CT_max_ in *B. impatiens* may depend on body size, but there has been mixed evidence for this effect in similar studies (electronic supplementary material, table S3).

While we did not observe air temperatures that exceeded thermal limits, bee *T*_th_ were often several degrees hotter than air temperatures (electronic supplementary material, figure S4), and did approach CT_max_, suggesting that bees foraging with full pollen loads on hot days face a stressful combination of conditions that they must counteract (figure 2). To do so, bees may adopt several strategies, each with potential costs at the individual or colony level. Bees may use physiological mechanisms to cool *T*_th_, such as regurgitating nectar for evaporative cooling [26]. However, this option may not be viable for pollen foragers with limited nectar stores [13]. Bees can also shunt excess heat to the head and abdomen [26], but this process may not provide sufficient cooling [28] and could result in heat injury to the head and abdomen, reducing forager efficiency or lifespan [29,30]. Bees may also choose to reduce the number or duration of pollen foraging trips [28,31] or shorten or shift their time windows for foraging [24,32]. Future research is needed to determine which of these strategies heat-stressed pollen foragers actually adopt and their consequences at the colony level.

## Data Availability

Raw data and code are available from the Dryad Digital Repository: https://doi.org/10.5061/dryad.0vt4b8h39 [33]. The data are provided in the electronic supplementary material [34].
